# Antitumoral Activity of Molecular Hydrogen and Proton in the Treatment of Glioblastoma: An Atypical Pharmacology?

**DOI:** 10.3390/brainsci13081168

**Published:** 2023-08-05

**Authors:** Luc Rochette, Geoffrey Dogon, Marianne Zeller, Yves Cottin, Catherine Vergely

**Affiliations:** 1 Pathophysiology and Epidemiology of Cerebro-Cardiovascular Diseases Research Unit (PEC2, EA 7460), University of Burgundy and Franche-Comté, UFR des Sciences de Santé, 7 Boulevard Jeanne d’ Arc, 21000 Dijon, France; geoffrey.dogon@u-bourgogne.fr (G.D.); marianne.zeller@u-bourgogne.fr (M.Z.); catherine.vergely@u-bourgogne.fr (C.V.); 2 Department of Cardiology, University Hospital of Dijon, 21000 Dijon, France; yves.cottin@chu-dijon.fr

**Keywords:** hydrogen, proton, pharmacology, glioblastoma

## Abstract

Antioxidants in cancer therapy have been a hot topic in the medical field for 20 years. Antioxidants are able to reduce the risk of cancer formation by neutralizing free radicals. Protons (H+) and molecular hydrogen (H2) interact in the cell and are essential in a wide variety of processes. The antioxidant, anti-inflammatory, and antiapoptotic effects of H2 have been studied in numerous experimental and clinical studies. Experimental data indicate that H2 is an antitumor agent in the treatment of glioblastoma (GBM). In vivo H2 inhalation could suppress the growth of GBM tumors, thereby extending the survival of mice with GBM. The sphere-forming ability of glioma cells was suppressed by hydrogen treatment. In addition, H2 treatment also suppressed the migration, invasion, and colony-forming ability of glioma cells. Proton therapy and proton beam radiotherapy offer some advantages over other modern conformal photon-based therapies when used in the treatment of central nervous system malignancies.

## 1. Introduction

Perturbations in cellular redox balance have been associated with aging mechanisms and increased risk of diseases such as cancer. A redox paradox has been described in cancer cells. The specificity of redox signaling depends on the generation and spatially regulated distribution of oxidants in the subcellular compartments of the cell. The use of antioxidants in cancer therapy has been a hot topic in the medical field for 20 years. It seems that antioxidants are able to reduce the risk of cancer formation by neutralizing free radicals. Anti-cancer drugs are cardiotoxic agents such as anthracyclines. Cardiotoxicity was attributed to oxidative stress induced by iron-based free radicals [1]. Resistance to cancer therapy is often observed as the metabolism and accumulation of iron have been disrupted in cancer. In this context, selective induction of ferroptosis could be an alternative anti-cancer strategy [2]. It is not clear what molecular mechanisms underlie the paradoxical role of oxygen and iron metabolism in countering carcinogenesis or improving sensitivity to cancer therapy [3]. Transition metal ions are key components of several biological processes, and their homeostasis is maintained within strict limits through tightly regulated mechanisms of redox- and non-redox-metal-induced free radical formation and their role in human diseases [4].

## 2. Hydrogen Homeostasis

The relationship between hydrogen homeostasis and transition metal ions is complex. Endogenous hydrogen (H2) in the human body is mainly produced by anaerobic bacteria in the gut and other organs by the reversible oxidation reaction of 2 H+ + 2 e − H2 [5]. Protons (H+) and molecular hydrogen (H2) interact in the cell and are essential in a wide variety of processes. H2 is considered a new type of natural antioxidant with a low reaction capacity with most biomolecules, which has potential therapeutic benefits [6]. Numerous studies in the literature have documented the importance of targeting nuclear factor erythroid-2 related factor 2 (NRF2)/antioxidant signaling and nuclear factor-kappa B (NF-κB) in the inflammatory response, as inflammation is a key factor in many pathological conditions such as cancer. H2 is now believed to influence intracellular parameters, including NRF2 and NF-κB. The displacement of NRF2 in the nucleus could lead to the regulation of gene expression involved in oxidative stress defense systems [7].

The antioxidant, anti-inflammatory, and antiapoptotic effects of H2 have been explored in numerous experimental and clinical studies [8,9,10,11]. The application of H2-enriched water prevented the production of UV-induced SAR and inhibited pro-inflammatory cytokine mRNAs for interleukin-6 and interleukin-1β [12]. H2 can exert an antiapoptotic effect by trapping ROS or regulating gene transcription, which can regulate endogenous apoptosis. An in vitro experiment showed that an H2-rich medium significantly inhibited ROS formation, maintained cell viability, and inhibited caspase-3 and caspase-9 in intestinal epithelial cells. In addition, H2 can activate the MAPK/HO-1 pathway to inhibit neuronal apoptosis and mitigate ischemic brain damage in neonatal mice [8,9].

Dysregulation of cellular pH is a well-known hallmark of malignancy. Hydrogen transport and cytoplasmic pH both play critical roles in the running of cell growth and proliferation, as well as tumorigenesis. In the cell, protons and molecular hydrogen are essential in an extensive diversity of processes. Recently, H2 has been studied in preclinical and clinical trials on several pathologies connected with oxidative stress and inflammatory process.

## 3. Hydrogen and Protons as Therapeutic Approaches in Cancer

The hypothesis of exploiting protons as an anti-cancer treatment was first suggested in 1946 by Robert R. Wilson, a physicist at the Harvard Cyclotron, who suggested that the unique physical characteristics of protons would be advantageous in the treatment of deep cancers. Today, proton therapy is emerging as a promising improvement over conventional external X-ray radiation therapy. X-rays and protons are forms of ionizing radiation that produces free radicals and ROS in cells [6]. Proton transport through the plasma membrane plays a central role in maintaining pH. Cells maintain intracellular pH (pHi) in a narrow range (7.1–7.2) by controlling proton pumps and membrane transporters. In humans, the maintenance of pH in the different cell compartments (intracellular and extracellular) is ensured by various regulatory systems. Ions use multiple pathways to enter the cytosolic environment. In this area, the bicarbonate ion (HCO3-) plays a fundamental role, and acido-basic homeostasis with HCO3- is critically regulated in various systems through different transporters. In addition, it is proposed that pHi plays an essential role in cancer metabolism, where an inverted pH gradient is a feature highlighted by extracellular acidosis and intracellular alkalization [10].

The acid-base balance is ordered by specific controls in cancerous and normal cells. Cancer cells exhibit unusual regulation of proton dynamics associated with regional hypoxia and increased glycolysis, resulting in extracellular acidity and intracellular alkalinity. The specific increase in cancer cells from proton extrusion outside the cell, which results in the creation of a proton gradient, occurs during the very early stages of neoplastic transformation. Oncogene-dependent transformation results in rapid alkalization of the cytoplasm, which has been considered a central factor in neoplastic transformation [11].

Studies have indicated that there is a crosstalk between the regulatory mechanisms of H2 on cancer, apoptosis, and autophagy [13]. In cancer biology, autophagy plays a dual role in tumor promotion and suppression and contributes to cancer cell development and proliferation [13]. In the cancer process, H2 plays a protective role by modulating autophagy in multiple diseases. H2 attenuates lung injury by inhibiting autophagy in alveolar epithelial cells of septic rats through inhibition of the p38 MAPK signaling pathway [14].

**Figure 1:**
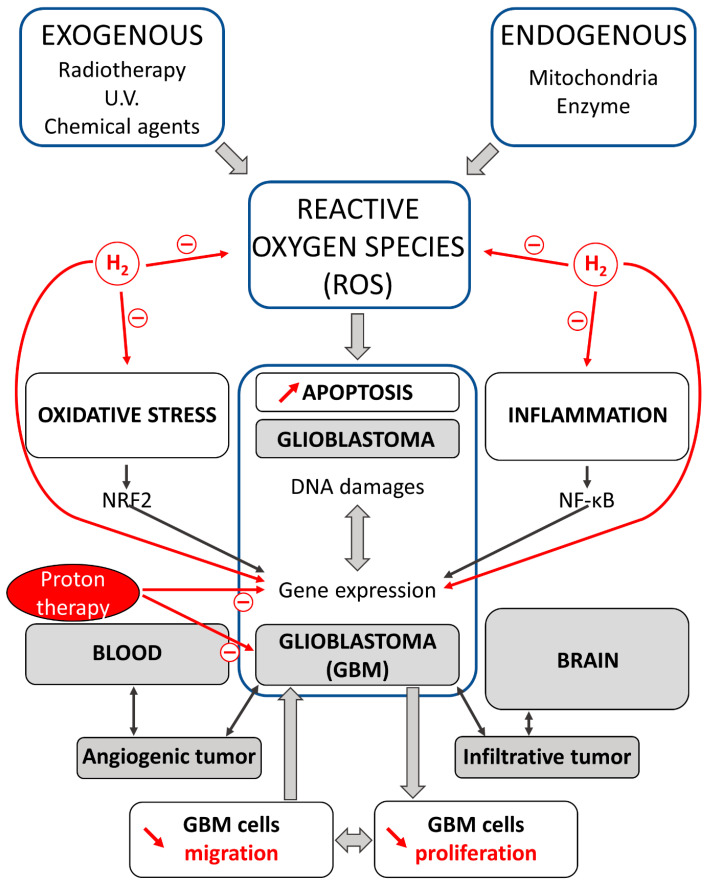
Effects of proton and H2 therapies on oxidative stress and inflammation in carcinogenesis. Reactive oxygen species (ROS) via oxidative stress and inflammation promote carcinogenesis in cells. A crosstalk exists between oxidative stress and inflammation; this interconnection is associated with the activation of NRF2 and NF-κB. Glioblastomas (GBMs) are intrinsic brain tumors that are thought to arise from neuroglial stem or progenitor cells. GBM displays high intra-tumor heterogeneity and an infiltrative nature. Proton and H2 therapies decrease cell migration and proliferation, and induce apoptosis in GBM cells. H2 and proton have demonstrated preventive and therapeutic effects in gliomas running a new view for the treatment of these malignant brain tumors.

High levels of ROS have been evaluated in cancer cell lines and implicated in disease progression and resistance to treatment. Increased levels of ROS are common to almost all tumor cells, and this has been suggested as a possible common focus for therapeutic advances [15]. Under these conditions, H2 and different types of H2 donors that reduce oxidative stress, and exert anti-inflammatory effects, represent a new therapeutic strategy in cancer treatment (Figure 1). Three methods are used for hydrogen administration: inhaling hydrogen gas, drinking hydrogen-rich water, or injecting hydrogen-rich saline. The rationale for the use of H2 in medicine is related to its antioxidant and anti-inflammatory properties in low concentrations through atypical pharmacology.

## 4. Hydrogen and Protons: Antitumor Agents in the Treatment of Glioblastoma (GBM)

Experimental results reveal that H2 is an antitumor agent in the treatment of glioblastoma (GBM). The invasive properties of GBM are a central issue for curative treatment if surgical resection is not feasible. GBM is the most common type of primary malignant brain tumor and is characterized by rapid proliferation, diffuse invasion into normal brain tissue, and high chemoresistance. Despite years of research into new therapies for GBM, current therapeutic strategies are insufficient to control the disease, as evidenced by relative survival rates of 37.4% at one year and 4.9% at five years [16]. Therefore, the advance of new treatment methods for GBM is imperative.

GBMs are intrinsic brain tumors that are thought to arise from neuroglial stem or progenitor cells. Over 90% of GBMs are isocitrate dehydrogenase (IDH)-wild-type tumors. The incidence increases with age, with men being more often affected. A new area of interest in the treatment of cancer, including GBM, is the repurposing of drugs already approved for other indications based on hypotheses of biochemical or metabolic characteristics that could confer sensitivity to glioma cells sensitivity to such drugs. The best part of clinical trial approaches focusing on GBM-intrinsic targets address oncogenic signaling via tyrosine receptor kinases, cell cycle control, and susceptibility to apoptosis induction [17].

In vivo experimental studies showed that H2 inhalation could repress the growth of GBM tumors, thereby prolonging the survival of mice with GBM. The in vivo studies were performed using an orthotopic rat glioma model and a subcutaneous mouse xenograft model. The animals inhaled H2 gas (67%) for 1 h twice a day. The sphere-forming ability of glioma cells was suppressed by hydrogen treatment. In addition, H2 treatment also suppressed the migration, invasion, and colony-forming ability of glioma cells [18].

As previously mentioned, H+ and H2 in the cell are critical in a wide variety of processes. Proton therapy (PT), or proton beam radiotherapy (PBRT), offers some advantages over other modern conformal photon-based therapies when used in the treatment of central nervous system (CNS) malignancies. Protons can produce disruptions in gene expression, alterations in signaling and function in the cell cycle, invasion, and angiogenesis. In cancer, they are able to limit progressive processes of invasion and migration. Protons are delivered with two different radiation techniques: passive scatter proton therapy (PSPT) or pencil beam scanning technique (PBS). Data specifically using PDT to treat recurrence in eight mostly high-grade glioma patients with a median reRT dose of 33 Gy and an initial dose of 55 Gy reported a median overall survival of 19.4 months with only two cases of uncomplicated radiological necrosis and no acute toxicity greater than class 2 [19]. There are still many challenges in the design and execution of PT trials [20].

In human models of GBM, recent results suggest that radiation-induced secretion of growth/differentiation factor 15 (GDF15) may contribute to tumor recurrence or resistance to treatment by mediating between brain cancer cells and endothelial cells [21]. Endogenous GDF15 is a ubiquitous cellular stress signal that can be produced and secreted by various cell types. Circulating levels are elevated in a range of disease states but also in response to exogenous and endogenous agents [22]. Among the endogenous modulatory proteins, β-arrestins are a group of adaptor proteins that regulate cell proliferation, promote invasion and cell migration. β-arrestins play primary roles in cancer invasion and metastasis through various signaling pathways. Interestingly, β-arrestin1 has been shown to be closely linked to the regulation of oxidative stress. β-arrestin1 interacts with GDF15 and facilitates the transport of the GDF15 precursor (pro-GDF15) to the Golgi system for cleavage and maturation [23].

Among the approaches engaged by cells to regulate intracellular pH, the Na/H exchanger protein 1 (NHE1) has been directly associated with cell transformation, invasion, and metastasis. Proton-bonded therapy for the treatment of malignant gliomas is emerging as a new therapeutic strategy. In this context, inhibition of NHE1 in gliomas acidifies tumor cells, while normal brain cells are not involved, running a new view for the treatment of these malignant brain tumors [24]. Drinking H2-enriched water reduces the volume and weight of endometrial tumors in a xenograft mouse model, inducing pyroptosis, an inflammatory programmed cell death involving the ROS/NLRP3/caspase-1 pathways. The pyrin domain containing 3 (NLRP3) inflammasome acts as a sensor that detects a wide range of pathological signals. This study shows that H2-stimulated endometrial cells can induce cellular activation of NLRP3 [25].

## 5. Conclusions

In conclusion, H2 and proton demonstrated preventive and therapeutic effects. The greatest benefits of using H2 or proton are the capacity to penetrate biological membranes and minimal undesirable effects. The molecular mechanisms underlying the paradoxical role of H2 and proton in countering carcinogenesis are not easily understood; the observed activities of H2 are dose-dependent. In some experiments, they appeared inconsistent and even illogical. H2 reduces oxidative stress, shows anti-inflammatory effects, and acts as a modulator of apoptosis. The elaboration of new schemes for the treatment of GBM is particularly imperative.

## Data Availability

Not applicable.
